# The Results of Five Years Dental Prophylaxis for the Employes of the Metropolitan Life Insurance Company

**Published:** 1922-01

**Authors:** Thaddeus P. Hyatt

**Affiliations:** Dental Director of the Metropolitan Life Insurance Company Clinic


### THE RESULTS OF FIVE YEARS DENTAL PROPHYLAXIS FOR THE EMPLOYES OF THE METROPOLITAN LIFE INSURANCE COMPANY

BY THADDEUS P. HYATT, D.D.S.
Dental Director of the Metropolitan Life Insurance Company Clinic

That regular periodical prophylactic treatments will bring about an improved condition of the mouth is shown by the figures secured during the years 1918, 1919 and 1920.

During the past five years, thousands of the employes of the Metropolitan Life Insurance Company have received two prophylactic treatments each year. Careful records have been made of mouth conditions. Cleanliness of the mouth has been classified under "Yes," and "No," or "Fair." Cases classified under "No" are those which show considerable amount of tartar deposits with inflammation of gum tissues. Those classified as "Fair" have slight deposits of tartar and slight gum inflammation, while those classified under "Yes" are practically free from tartar or gingivitis. It should be understood that this classification was made solely from the viewpoint of hygiene or whether the patient's health might be affected by mouth conditions. Therefore, those classified under "Yes" must not be understood as being perfectly clean. There may be found stains either from tobacco or other causes, but this was not expected to influence the health condition of the mouth nor of the patient.

Figures secured under these classifications should prove of value and of interest.

All employes who first came to the Dental Division in 1915, and who have continued to come twice each year, are called Class 1; those who first came in 1916 are called Class 2; those in 1917, Class 3; in 1918, Class 4; in 1919, Class 5; and in 1920, Class 6.

It was in 1918 when we first ascertained the percentages of "Yes," "No" or "Fair" for the four classes we had at that time.

PERCENTAGE TABLE OF "YES," "NO" AND "FAIR" IN 1918

| | Yes | No | Fair |
|---|---|---|---|
| Class 1 started in 1915........ | 27% | 25% | 48% |
| Class 2 started in 1916........ | 25% | 27% | 48% |
| Class 3 started in 1917........ | 18% | 29% | 53% |
| Class 4 started in 1918........ | 10% | 50% | 40% |

These figures show that 27 per cent. of Class 1 were "Yes"; in Class 2, 25 per cent.; Class 3, 18 per cent.; and in Class 4 only 10 per cent. The class that had been coming regularly twice each year for the longest period showed the highest per cent. of "Yes" and the balance of the classes showed their respective standings according to the length of time they had received these treatments.

It is interesting to note that the youngest class at that time—namely, the fourth class—showed 50 per cent. under "No," while the first class showed only 25 per cent.

PERCENTAGE TABLE OF "YES," "NO" AND "FAIR" MADE IN 1918, 1919 AND 1920

| | Yes | | | No | | | Fair | | |
|---|---|---|---|---|---|---|---|---|---|
| | 1918 | 1919 | 1920 | 1918 | 1919 | 1920 | 1918 | 1919 | 1920 |
| Class 1 started in 1915.... | 27% | 33% | 39% | 25% | 9% | 6% | 48% | 58% | 55% |
| " 2 " " 1916.... | 25 | 33 | 43 | 27 | 9 | 7 | 48 | 58 | 50 |
| " 3 " " 1917.... | 18 | 35 | 42 | 29 | 9 | 6 | 53 | 56 | 52 |
| " 4 " " 1918.... | 10 | 27 | 40 | 50 | 14 | 9 | 40 | 59 | 51 |
| " 5 " " 1919.... | | 21 | 33 | | 27 | 12 | | 52 | 55 |
| " 6 " " 1920.... | | | 13 | | | 31 | | | 56 |

In examining this table, we notice the interesting fact that Class 1 in 1918 showed 27 per cent. under "Yes" and that this is increased to 33 per cent. in 1919 and 39 per cent. in 1920.

We will also notice that in 1918, Class 1 had 25 per cent. under "No" and that this is reduced to 9 per cent. in 1919 and to 6 per cent. in 1920.

In Class 2, we find the same marked improvement. In 1918, 25 per cent. of this class had "Yes" mouths. This is increased to 33 per cent. in 1919 and to 43 per cent. in 1920. Studying this class under the heading of "No," we find in 1918 there is 27 per cent., which was reduced to 9 per cent. in 1919 and to 7 per cent. in 1920.

We will notice this same improvement in each of the different classes. In studying these figures under the heading of "Fair," we notice an interesting fact. In 1919, Class 1 had 58 per cent. "Fair." In 1920, this is reduced to 55 per cent., or a loss of 3 per cent. We will also notice there is a reduction under the heading "No." In 1919 there was 9 per cent. and in 1920 6 per cent., or a loss of 3 per cent. This makes a total loss of 6 per cent. under "No" and "Fair."

If we study the figures under "Yes" we will find that there has been an increase of 6 per cent. from 1919 to 1920. This shows that 3 per cent. was transferred from the "No" to the "Yes" and 3 per cent. from the "Fair" to the "Yes"; or, of course, it would be possible that the entire 6 per cent. promotion took place from the "Fair" to the "Yes" and 3 per cent. from the "No" into the "Fair."

Studying Class 2 under the heading "Fair," we find 58 per cent. in 1919 and only 50 per cent. in 1920. This class, under the heading "No" shows 9 per cent. in 1919 and 7 per cent. in 1920. This makes a total loss of 10 per cent. in these two classes. This is found to be accounted for under the heading "Yes," because here we find the increase under "Yes" goes from 33 per cent. in 1919 to 43 per cent. in 1920, or an increase of 10 per cent.

The improvement in Class 1 from 1918 to 1919 is shown as follows: We find a gain of 6 per cent. under "Yes," a gain of 10 per cent. under "Fair," which amounts to 16 per cent. We will notice that is a loss of 16 per cent. under "No."

It is also interesting to note all of the classes 1, 2, 3 and 4 show an increased per cent. under "Fair" between 1918 and 1919, but all show a loss between 1919 and 1920.

but in every case the gain under "Yes" equals the loss under "Fair" plus the loss under "No."

Class 5 also shows an increase under "Fair" for its first two years and it will be interesting to note if in its third year it will respond to the same results as have the first four classes.

These figures secured from over 26,000 cleansing operations should be sufficient to prove that regular periodical prophylactic treatment does bring about an improved condition of the mouth.—*Items of Interest.*

